# A prospective randomized study, use of closed suction drainage after revision hip arthroplasty may lead to excessive blood loss

**DOI:** 10.1038/s41598-022-05023-2

**Published:** 2022-01-18

**Authors:** Paweł Bartosz, Dariusz Grzelecki, Sławomir Chaberek, Marcin Para, Wojciech Marczyński, Jerzy Białecki

**Affiliations:** 1 Orthopedic Department, Centre of Postgraduate Medical Education, Konarskiego 13, 05-400 Otwock, Poland; 2 Department of Orthopedics and Rheumoorthopedics, Centre of Postgraduate Medical Education, Otwock, Poland; 3 Gruca Orthopedic and Trauma Teaching Hospital, Otwock, Poland

**Keywords:** Randomized controlled trials, Orthopaedics

## Abstract

Suction drainage after primary total hip arthroplasties (THA) offers no benefits. Revision hip arthroplasties (RHA) are more demanding procedures and associated with greater blood loss compared to primary cases. There is still a lack of literature regarding the application of drainage in RHA. A total of 40 patients who underwent RHA were included in this prospective study. Simple randomization with an allocation ratio 1:1 was performed. Primary outcomes: total blood loss, hemoglobin drop, joint hematoma size in USG, infection. Secondary outcomes: blood transfusion rate, soft tissue hematomas, C-reactive protein levels, Visual Analogue Scale before and on 3rd day after surgery, Harris Hip Score before and 6 weeks after surgery. An intention to treat analysis was performed, with a 2-year follow up. Statistically significant differences between groups was in blood loss: drainage 1559.78 ml, non-drainage 1058.27 ml, (p = 0.029) and hemoglobin level on 1st day after surgery: drainage 10.58 g/dl, non-drainage 11.61 g/dl (p = 0.0496). In terms of the other analyzed parameters, statistical differences were not found. Our study revealed that the use of suction drainage may lead to higher blood loss in the early postoperative period. Further studies are needed to evaluate our results.

## Introduction

Current literature for primary total hip arthroplasties (THA) shows no benefits of closed suction drainage after surgery^1,2^. Revision hip arthroplasties (RHA) compared to primary procedures are more aggravating for both patients and surgeons. Different procedures such as mobile components exchange, partial reimplantation of a cup or stem, and the most difficult cases of total prosthesis reimplantation are performed during the RHA. An extended surgical approach, soft tissue damage and bone defects, longer time of surgery, and the need to use more complex implants for acetabulum or modular stems are the main features of RHA that influence on higher complication rate including perioperative blood loss^3–5^.

Blood loss after surgery is one of the major problems of RHA. In these cases, drainage application prevents hematoma organization in the joint space and leads to its evacuation, which may subsequently cause a significant hemoglobin and hematocrit drop. To decrease blood loss and amount of transfusion several methods such as autologous blood transfusion, and intraoperative blood savers can be used^6,7^. Additionally, in recent years tranexamic acid or aminocaproic acid have found the application in both THA and RHA for that purpose. The administration of antifibrinolytic agents in different doses and protocols in RHA decrease perioperative blood loss and the need for blood transfusion without increasing deep venous thrombosis (DVT) and pulmonary embolism (PE) events^8,9^.

Blood transfusion is an independent factor of septic complication after THA (0.5–2%) and RHA (10%)^10,11^. However, the tamponade effect caused by hematoma organization may decrease bleeding into the joint space and the need for a transfusion, but potentially increase periprosthetic joint infection incidence^12^. Therefore, there is still no sufficient evidence to determine the use of suction drainage in extensive orthopedic surgical procedures such as RHA^13^. Moreover, a joint hematoma can lead to other complications such as prolonged wound leakage, joint stiffness, flexion contracture, that influence negatively on clinical and functional outcomes and provoke the need for further surgical procedures^14^.

The aim of this study was to evaluate the impact of suction drainage in terms of blood loss, hemoglobin drop, intra-articular size of the hematoma, and infection incidence after RHA.

## Methods

This study received from Bioethical Committee at the Centre of Postgraduate Medical Education in Warsaw approval number 13/PB/2016 on 9 March 2016 and was successfully retrospectively registered at ClinicalTrial.gov with identification number: NCT04486040 (registration date 24/07/2020). All methodology was performed according to above institution guidelines.

A total number of 40 consecutive patients who underwent RHA and signed their informed consent for participation were included in this prospective study and achieved final follow-up. All patients were treated in Orthopedic Department, Centre of Postgraduate Medical Education in Otwock. The inclusion criteria were set as RHA performed due to aseptic implant loosening (cup, stem or both elements). Septic implant loosening, primary or secondary coagulopathy, chronic inflammatory diseases (such as rheumatoid arthritis, ankylosing spondylitis), concomitant malignancy, renal or hepatic failure, as well as thromboembolism in the past were stated as exclusion criteria.

Simple randomization with an allocation ratio of 1:1 was performed. Envelopes with information on drainage use (or not) were drawn and opened at the end of surgery by the anaesthesiologist team. Up to this moment neither the patient or orthopaedic surgeons knows what type of intervention will be applied. Unblinded investigator collected postoperative data. Patients were typically prepared for revision surgery. Every patient got thromboprophylaxis with the use of ultra-low molecular weight heparin (ULMWH) before surgery and this was continued for 35 days. All patients received antibiotic prophylaxis and tranexamic acid (one dose (15 mg/kg) 15 min before the surgery and a second dose 6 h after). In all cases between 3 to 6 samples (synovial fluid, periprosthetic tissues and implant for sonication) were taken for microbiological culturing. All patients got empiric antibiotic therapy until receipt of results of intraoperative microbiological specimens. However, no prior infection was confirmed. If drainage was used, it was removed after 24 h. All patients were walking with two crutches on the first day after surgery. On the third day after the operation, all patients underwent ultrasonography (USG) for the measurement of the fluid in the joint and the presence of haematoma in soft tissue. For the USG all patients were lined up in the same position^15^. If soft tissue haematoma was confirmed, depending on indications, punction and evacuation was performed. C-reactive protein levels (CRP), haemoglobin, haematocrit were measured in the early postoperative period on the first and third day, and at the first control outpatient visit after 6 weeks. All data were collected by the primary investigator and all USG exams were performed by one examiner. We assessed wound healing as dry or excluded dressing after 3 days post-surgery.

Primary outcomes were blood loss after surgery, joint haematoma, haemoglobin level after surgery, and infection. To assess “hidden” blood loss the Gross formula was used^16^. Signs of infection like wound healing problems, fever, hip pain, CRP increase, were noted directly after surgery and at all check visits. Secondary outcomes were set as soft tissue haematomas, CRP levels, need for blood transfusion after RHA. Patients’ clinical and functional outcomes were measured with Harris Hip Score (HHS) before surgery and 6 weeks after and Visual Analogue Scale (VAS) before surgery and on 3rd day after surgery (range 1–10).

Intention-to-treat analysis were performed. Analyses were performed using Statistica 13.1 for Windows (StatSoft, Inc., STATISTICA for Windows, Tulsa, OK). Variables were reported as mean, standard deviation, range (min/max). The analysed groups were compared using descriptive statistics, cross-tabulation tables, and non-parametric statistics analysis. For comparison between groups the T-Test for Independent Samples for normally distributed data was used. The Mann–Whitney U test for non-normally distributed data was used. Categorical data were compared by means of the Chi-squared test, M-L Chi-square test and Yates Chi-square test. Tests results were defined as statistically significant with p < 0.05 (p values < 0.05 were considered statistically significant).

After including 20 patients in each group the research was unblinded and preliminary statistical analysis was done. We revealed significant differences in terms of total and percentage blood loss, and haemoglobin concertation on the first postoperative day as primary investigated outcomes. Thus, the decision to stop the trial and further randomization to reduce the risk to patients was made.

## Results

Finally, 40 patients were included in the analysis, 20 with drainage and 20 without drainage application. The mean age was 60.6 (± 13.18) years. The group consists of 23 women and 17 men. The average Body Mass Index (BMI) was 27.3 (± 5.13) kg/m^2^. Pre-operative diagnosis and type of procedure distribution between groups were shown in Table 1. We started our study in March 2016 and the last patient was qualified in February 2018. Follow-up in both groups was at least 2 years after surgery (mean 3.4 years, range from 2.2 to 4.1 years), and reinfection was analysed at the final outpatient visit.

**Table 1:** Type of procedure and preoperative diagnosis.

	Drainage (n = 20)	Non-drainage (n = 20)	p-value
**Type of revision**			
Cup	5	7	0.31*
Stem	2	0	
Both elements	13	13	
**Diagnosis**			
Aseptic loosening	10	9	0.75**
Post-infection	10	11	
Girdlestone hip	9	9	
Spacer exchange	1	2	

*Chi^2^ Test (3 × 2).

**Chi^2^ Test (2 × 2).

The statistical analysis did not reveal any significant differences between groups with respect to demographic and perioperative parameters (Table 2).

**Table 2:** Clinical and demographic data between the study groups. Continuous variables were presented as means (± SD).

	Drainage (n = 20)	Non-drainage (n = 20)	p-value
Males/females	9/11	8/12	0.75*
Age (years)	59.6 (± 10.8)	62.1 (± 14)	0.52**
BMI (kg/m^2^)	27.7 (± 5.6)	26.9 (± 4.4)	0.63**
Operating time (minutes)	148 (± 43.3)	138.8 (± 43.9)	0.51**
Hospital stay (days)	11.3 (± 3.6)	12 (± 4.5)	0.64**
Total blood volume (Nadler’s formula^17^) (mL)	4763.6 (± 946)	4631.5 (± 899)	0.65**
APTT	25.9 (± 4.9)	25.3 (± 4.2)	0.66**
PT	13.5 (± 1.2)	13.4 (± 1.4)	0.76**
INR	1.09 (± 0.08)	1.09 (± 0.1)	0.97**
**Fixation type**			0.26*
Cemented	3	6	
Uncemented	17	14	

*Chi^2^ Test (2 × 2).

**Student T test.

*BMI* Body Mass Index, *APTT* activated partial thromboplastin time, *PT* prothrombine time, *INR* international normalized ratio.

Statistically significant differences in total blood loss, including intraoperative and hidden, between groups were found (p = 0.029). In the drainage group, the mean blood loss was 1559.78 (± 734) mL, and in the non-drainage group was 1058.27 (± 623) mL. The haemoglobin level on the first day after surgery in the no-drainage group was significantly higher than in the drainage group (11.61 vs. 10.58 mg/dl; p = 0.0496). For the other anaemia related parameters (haematocrit value on the first postoperative day and haemoglobin concentration on the third postoperative day) statistically significant differences between groups were not observed (p = 0.116 and p = 0.181 respectively). Haemoglobin concentrations between the first and third postoperative days (p = 0.965) and joint haematoma size in the USG examination on the third day (p = 0.328) were not significantly different between the examined groups. In the drainage group, in 6 cases we noticed soft tissue haematomas compared to 5 cases in the non-drainage group. Additionally, there were no significant differences between the drainage and non-drainage groups in the number of transfused blood units (37 vs 20; p = 0.13) and number of patients who required transfusion (13 vs 8; p = 0.11).

Only one infection in the 2 years follow up was noted in the drainage group, and no such complication in the non-drainage group was confirmed.

For patients’ reported outcomes, we checked pain with VAS and function with the HHS scale. Both scales were taken before surgery and VAS on the third day after surgery, then HHS 6 weeks after at the first control visit. In both parameters statistical differences between groups were not found, p = 0.603 and p = 0.589, respectively (Table 3).

**Table 3:** Primary and secondary outcomes.

	Drainage (n = 20)	Non-drainage (n = 20)	p-value
Total blood loss 1st day (mL)	1641 (± 913)	1089.3 (± 619)	0.03**
Percentage blood loss 1st day (%)	34.1 (± 16.7)	25.6 (± 19.3)	0.039***
Preoperative Hb concentration (mg/dl)	13.9 (± 1.3)	14.1 (± 1.8)	0.57**
Hb 1st day (g/dL)	9.8 (± 1.5)	11 (± 2.1)	0.037**
Hb 3rd day (g/dL)	8.3 (± 1.4)	9.2 (± 1.9)	0.11**
Haematoma size (mm)	17.7 (± 11.3)	14.9 (± 11.4)	0.8***
Soft tissue haematomas (no.)	6	5	0.72*
Infection (no.)	1	0	0.99*
30-days readmission	1	1	1*
CRP 1st day (mg/L)	164.2 (± 82.5)	204.1 (± 69.4)	0.11**
CRP 3rd day (mg/L)	112.9 (± 53.7)	173.9 (± 94.6)	0.08***
Wound leakage at 3rd day (no.)	12	7	0.11*
VAS before	3.8 (± 1.7)	3.2 (± 1.3)	0.17**
VAS 3rd day	3.6 (± 1.6)	3.9 (± 1.5)	0.61**
Preoperative HHS	45.4 (± 21.8)	46.8 (± 14.8)	0.81**
HHS 6 weeks	65 (± 19.1)	61.9 (± 14.2)	0.56**
Total no. of blood units	37	20	0.13***
Patients with blood transfusion (no.)	13	8	0.11*
Units of blood per patient	1.95	1.05	

Significant values are in bold.

*Chi^2^ Test (2 × 2).

**Student T test.

***Mann–Whitney U test.

*Hb* haemoglobin, *CRP* C-reactive protein, *VAS* Visual Analogue Scale, *HHS* Harris Hip Score, *U.* units, *No.* number.

## Discussion

Strict recommendations for the use of suction drainage in total joint arthroplasties are currently unavailable. Despite this fact, there are many scientific reports emphasizing that non-draining protocols are safe and effective in THA, there is still not sufficient evidence to support or discourage the routine use of suction drainage in RHA. We found statistically significant lower volume of blood loss after surgery and haemoglobin drop at day 1 after surgery in no-drainage group. Fichman et al. show that in RHA without drainage, postoperative haemoglobin drop, hospital stay, and the number of transfusions were lower compared to the control group^13^. Despite this study, the number of transfused blood units did not differ significantly in our research—in fact almost two times more blood units had to be transfused in the drainage group.

In our study, we also checked the level of joint haematoma after surgery, which could lead to infection, limited range of motion and pain. No statistical differences were found, but unexpectedly in the non-drainage group average level of joint fluid on the endoprosthesis neck after surgery was 14.8 mm and in the drainage group 17.9 mm. We did not found studies describing the volume of haematoma in the joint space after RHA, especially comparing drainage and non-drainage patients. To reduce bleeding and transfusion rate, tranexamic acid was administered in all patients without contraindications following the current literature evidence in RHA^18,19^. It decreases blood loss and the number of blood transfusions required without increasing the risk of deep vein thrombosis and pulmonary embolism.

Parvizi et al. show that excessive anticoagulation therapy leads to wound problems, and higher infections rate^20^. In the authors’ country, due to legal conditions, shorter anticoagulation therapy or only aspirin use in hip replacement is not possible. Despite that, a higher incidence of wound problems or haematoma formation in our population was not confirmed. Wound healing problems are more frequent in RHA^21^. Our results stay in line with Ashraf et al., who showed that in RHA suction drainage did not protect against wound oozing and haematoma formation^22^. Moreover, we did not find statistical differences in wound leakage and other wound healing problems between groups.

Both wound healing problems and blood transfusions are well-known factors increasing infection probability^20,23^. The infection rate in revision hip arthroplasty varies from 7.9 to 13% in RHA^24,25^. In our cohort infection, at the 2 years follow up, was diagnosed in 1 patient from the drainage group (2.6%). We also checked CRP levels after surgery, which are inflammation markers and when increasing could be periprosthetic joint infection predictors. Fink et al. and Stambough et al. found that CRP is not a good predictor for infection, because low-grade infections could persist with normal values of CRP and in the rheumatology patients CRP could be elevated^26,27^. In our study, we did not observe significant differences between groups in CRP levels after surgery.

In addition HHS 6 weeks after surgery showed no differences between groups. Potentially in the non-drainage group, joint haematoma could have given more pain, but in VAS we did not observe differences between groups as well.

There are several limitations to this research. First, it was a single-centre study and therefore may be subject to selection bias. For this reason, we instituted strict inclusion and exclusion criteria, also simple randomisation was performed to reduce selection bias. Another limitation was the small groups included in this study. We checked results after 20 patients in each group and when statistically significantly higher blood loss in drainage was found we stopped the trial. Further continuing the study could have lead to greater risk of complications in examined patients. Including patients with a high range of revision surgeries was also a serious limitation, but after randomization, allocation of procedures between groups was similar. Multi-center studies are needed to validate our findings and increase the power of their results.

In conclusion we recommend not use closed suction drainage after revision hip arthroplasty. According to our results drainage could lead to greater total and percentage blood loss, and hemoglobin drop on the first day after surgery.
